# CRISPR/Cas9-mediated miR-21 editing in high-grade urothelial carcinoma cells and its biological effects

**DOI:** 10.1007/s11033-026-12129-7

**Published:** 2026-06-19

**Authors:** Maria Carolina Yi Lin Lee, Juliana Alves Camargo, Giovana Caetano Vilas Boas, Karina Serafim Silva, Luana Pereira Silva, Carolina Mie Mioshi, Ruan Pimenta, Iran Amorim Silva, Katia Ramos Moreira Leite, William Carlos Nahas, Sabrina T dos Reis

**Affiliations:** 1 Laboratory of Medical Investigation (LIM55), Urology Department, University of São Paulo Medical School, Av. Dr. Arnaldo 455, 2° floor, room 2145 - Cerqueira Cesar, São Paulo, Brazil; 2 Precision Immunology Institute, Department of Immunology and Immunotherapy, and Tisch Cancer Institute, Icahn School of Medicine at Mount Sinai, New York, United States; 3 Uro-Oncology Group, Urology Department, Institute of Cancer State of São Paulo (ICESP), São Paulo, Brazil; 4 Moriah Institute of Science and Education (MISE), Hospital Moriah, São Paulo, Brazil

**Keywords:** Bladder cancer, CRISPR/Cas9, miR-21

## Abstract

**Background:**

Urothelial carcinoma, the predominant form of bladder cancer, represents a global public health challenge due to its high rates of recurrence and progression. At the molecular level, microRNA-21 (miR-21) has been characterized as an “oncomir” because of its ability to negatively regulate tumor suppressor genes, thereby promoting tumor survival and progression. In this context, the CRISPR/Cas9 system has emerged as a precise genome-editing tool.

**Objective:**

To investigate the biological effects of miR-21 modulation using CRISPR/Cas9-mediated genome editing in the T24 high-grade invasive urothelial carcinoma cell line.

**Methods:**

The CRISPR/Cas9 system was delivered as a ribonucleoprotein (RNP) complex. Editing efficiency was assessed using quantitative reverse transcription PCR (RT-qPCR). Functional effects were evaluated through gene expression assays, cell migration assays, as well as Matrigel invasion assays. The presence of the Cas9 protein was confirmed by immunofluorescence.

**Results:**

CRISPR/Cas9 treatment targeting miR-21 showed a trend toward reduced miR-21 expression (p = 0.0563), although this did not reach statistical significance. A statistically significant increase in MASPIN (p < 0.0001) and PDCD4 (p = 0.0239), as well as a trend toward increased PTEN expression (p = 0.055), was observed following treatment. Functionally, a significant reduction in the migratory capacity of edited cells was observed after 48 h (p = 0.0334). The presence of Cas9 was successfully confirmed in transfected cells.

**Conclusion:**

These findings suggest that CRISPR/Cas9-mediated modulation of miR-21 may influence tumor suppressor pathways and reduce the migratory potential of urothelial carcinoma cells.

**Supplementary Information:**

The online version contains supplementary material available at 10.1007/s11033-026-12129-7.

## Introduction

Bladder cancer (BC) represents a significant global public health concern. According to GLOBOCAN 2020 estimates, more than 614,000 new cases and approximately 220,000 deaths were reported worldwide, maintaining an upward trend observed in recent years [1]. The disease exhibits a clear sex-related disparity, with a higher incidence in men, as well as a heterogeneous geographic distribution, being more prevalent in regions with a high Human Development Index (HDI), such as Europe and North America, where smoking and occupational exposure to carcinogens are more common [2, 3]. In Brazil, approximately 11,370 new cases per year were estimated for the 2023–2025 triennium, also with a predominance in males [4]. Smoking is the primary risk factor, followed by occupational exposure to chemical compounds such as aromatic amines and hydrocarbons. However, biological factors also appear to contribute to the higher incidence in men, including differences in xenobiotic metabolism and hormonal signaling, while female hormones may exert a protective effect [5–10].

From a clinical perspective, staging is based on the Tumor–Node–Metastasis (TNM) system, with most cases classified as non–muscle-invasive bladder cancer (NMIBC). Despite a favorable initial prognosis, these lesions present high recurrence rates. In contrast, muscle-invasive bladder cancer (MIBC), present in approximately 25% of patients, is associated with a higher risk of progression and poorer survival. Diagnosis is confirmed by histopathological analysis obtained through cystoscopy or transurethral resection of bladder tumor (TURBT), a procedure that also plays an initial therapeutic role. Treatment varies according to disease stage, including intravesical Bacillus Calmette–Guérin (BCG) therapy for non-invasive cases and radical cystectomy combined with chemotherapy for advanced disease. More recently, immunotherapy with checkpoint inhibitors has expanded therapeutic options, although responses remain limited in a subset of patients [11, 12].

The clinical course and treatment response of bladder cancer are highly variable, indicating that the molecular mechanisms underlying tumor progression are not yet fully understood. In this context, microRNAs (miRNAs) have gained attention due to their role in regulating gene expression, particularly at the post-transcriptional level through translational repression or degradation of target mRNAs [13]. Among them, miR-21 is one of the most consistently described miRNAs in the literature as an “oncomiR”, frequently overexpressed in various tumor types, including urothelial carcinoma [14–18]. Its activity is associated with the inhibition of tumor suppressor genes such as MASPIN (mammary serine protease inhibitor), PDCD4 (programmed cell death protein 4), and PTEN (phosphatase and tensin homolog), promoting increased cell proliferation, resistance to apoptosis, and enhanced invasive capacity [16–27].

Despite the well-established role of miR-21, few studies have directly evaluated the effects of its loss of function. Genome-editing technologies, such as the CRISPR (Clustered Regularly Interspaced Short Palindromic Repeats)/Cas9 (CRISPR-associated protein 9) system, currently enable a more precise assessment of miRNA loss-of-function effects [28–32]. Therefore, this study aimed to evaluate the effects of miR-21 editing using CRISPR/Cas9 in high-grade urothelial carcinoma cells, focusing on changes in tumor suppressor gene expression and biological processes involved in tumor progression.

## Methods

### Cell culture

The human T24 cell line, derived from high-grade invasive urothelial carcinoma of the bladder, was obtained from the American Type Culture Collection (ATCC) and authenticated prior to use (Annex 1). Experiments were performed using cells between passages 5 and 15. According to Resolution No. 510/2016 of the Brazilian National Health Council, the use of established in vitro cell lines does not require approval by an ethics committee. Cells were cultured in McCoy’s 5 A Modified medium supplemented with 10% fetal bovine serum (FBS) and 1% antibiotic–antimycotic solution (Sigma Co., St. Louis, MO, USA). Cultures were maintained in an incubator at 37 °C in a humidified atmosphere containing 5% CO₂, with medium renewal every 72 h.

### Transfection of the T24 cell line with RNP complex

Ribonucleoprotein (RNP) complex delivery was performed in 12-well plates containing 1.25 × 10^3^ cells per well. The single guide RNA (sgRNA) used to target miR-21 was designed using the CRISPRpick software developed by the Broad Institute. The selected sequence (sgRNA: ATCTCATGGCAACACCAGTCG) was purchased from Synthego.

For transfection, two mixtures were prepared separately. In tube 1, 6.25 µL of Opti-MEM medium, 0.33 µL of guide RNA, 0.25 µL of Cas9 enzyme, and 0.25 µL of Lipofectamine™ Cas9 Plus (Thermo Fisher Scientific) were combined, followed by mixing and incubation at room temperature for 10 min. In tube 2, 6.25 µL of Opti-MEM and 0.38 µL of Lipofectamine™ CRISPRMAX were added and incubated at room temperature for 5 min. Subsequently, the contents of tube 2 were added to tube 1 and incubated for 5 min to allow formation of the transfection complex. Next, 86.29 µL of complete McCoy’s medium were added, resulting in a final mixture volume of 100 µL. This solution was added to wells containing 900 µL of complete medium, yielding a final volume of 1 mL per well. Cells were incubated overnight at 37 °C in a humidified atmosphere with 5% CO₂, and the medium was replaced the following day.

Experiments were performed using independently prepared cell cultures, employing T24 cells transfected with a CRISPR/Cas9 RNP complex containing an sgRNA targeting miR-21, along with a scramble control group transfected with the same CRISPR/Cas9 system containing a non-targeting sgRNA sequence. Each experimental condition was analyzed using technical replicates in multiple wells. When necessary, previously transfected cells were subjected to retransfection to maintain modulation effects.

### RNA extraction and cDNA synthesis

Total RNA was extracted separately for each experimental condition in each repetition, without pooling between distinct experiments. Extraction was performed using the miRVana™ kit (Thermo Fisher Scientific), according to the manufacturer’s protocol, including cell lysis, phenol–chloroform extraction, and silica column purification. RNA concentration was determined by fluorometry using the Qubit™ 4 system (Thermo Fisher Scientific), while RNA purity was assessed by spectrophotometry. Samples with an A260/A280 ratio between 1.8 and 2.0 were considered acceptable. RNA integrity was additionally evaluated by agarose gel electrophoresis, confirming preservation of ribosomal RNA bands.

For miRNA analysis, cDNA was synthesized using the TaqMan™ MicroRNA Reverse Transcription kit (Applied Biosystems), with specific stem-loop primers, under the following conditions: 16 °C for 30 min, 42 °C for 30 min, and 85 °C for 5 min. For mRNA analysis, cDNA synthesis was performed using the High-Capacity cDNA Reverse Transcription kit (Applied Biosystems), employing random primers.

### RT-qPCR and gene expression analysis

Gene expression quantification was performed using the ABI 7500 Fast Real-Time PCR System (Applied Biosystems) with TaqMan™ Universal PCR Master Mix. The TaqMan™ assays used for each target are described in Supplementary Table 1. Reactions (10 µL) were conducted in technical duplicates under the following conditions: 95 °C for 10 min, followed by 40 cycles of denaturation and annealing/extension at 60 °C.

Relative expression levels were calculated using the comparative 2^−ΔΔCt^ method, as previously described by Livak and Schmittgen [33], with B2M (for mRNA) and RNU48 (for miRNA) used as endogenous controls. Normalization was performed relative to the control group.

## Functional assays

### Migration assay

Migration assays were performed using the wound-healing method as previously described by Liang et al. [34]. Cells at approximately 95% confluence were subjected to a scratch (“wound”) using a sterile 200 µL pipette tip. Images were acquired at 0, 24, and 48 h using an inverted microscope (Nikon). The migration rate was quantified as the percentage of wound closure using NIS-Elements D 3.1 software (Nikon).

Cell migration percentage was determined based on changes in wound area over time using the following equation: Cell migration (%) = [(D_initial − D_final) / D_initial] × 100, where D_initial corresponds to the wound distance at time 0 and D_final to the distances measured at 24 and 48 h.

### Invasion assay

Invasive potential was evaluated using Matrigel-coated Boyden chambers as previously described [35]. After serum starvation for 24 h, 5 × 10⁴ cells suspended in serum-free medium were seeded into the upper chamber, while the lower compartment contained medium supplemented with 10% fetal bovine serum as a chemoattractant. After 48 h, invasive cells were fixed with 4% paraformaldehyde, stained with 0.2% crystal violet, and quantified by counting the cells on the lower surface of the membrane under 200× magnification, allowing determination of the number of invasive cells and colonies.

### Immunofluorescence

Transfection efficiency was evaluated 72 h after treatment with the CRISPR/Cas9 system. Cells were fixed with 4% paraformaldehyde, permeabilized with 0.1% Triton X-100, and incubated with a primary anti-Cas9 antibody (MA5-23519; Thermo Fisher Scientific), followed by a fluorophore-conjugated secondary antibody. Nuclei were stained with DAPI. Images were acquired using a fluorescence microscope for qualitative assessment of Cas9 protein expression.

### Statistical analysis

All experiments were independently performed at least three times, and each condition was analyzed in technical duplicates. Statistical analyses were performed using GraphPad Prism 9.0 software. Data normality was assessed using the Shapiro–Wilk test. Comparisons between two groups were conducted using the Student’s t-test or the Mann–Whitney U test, depending on data distribution. A p-value < 0.05 was considered statistically significant.

## Results

### Modulation of miR-21 by CRISPR/Cas9

**Fig. 1:**
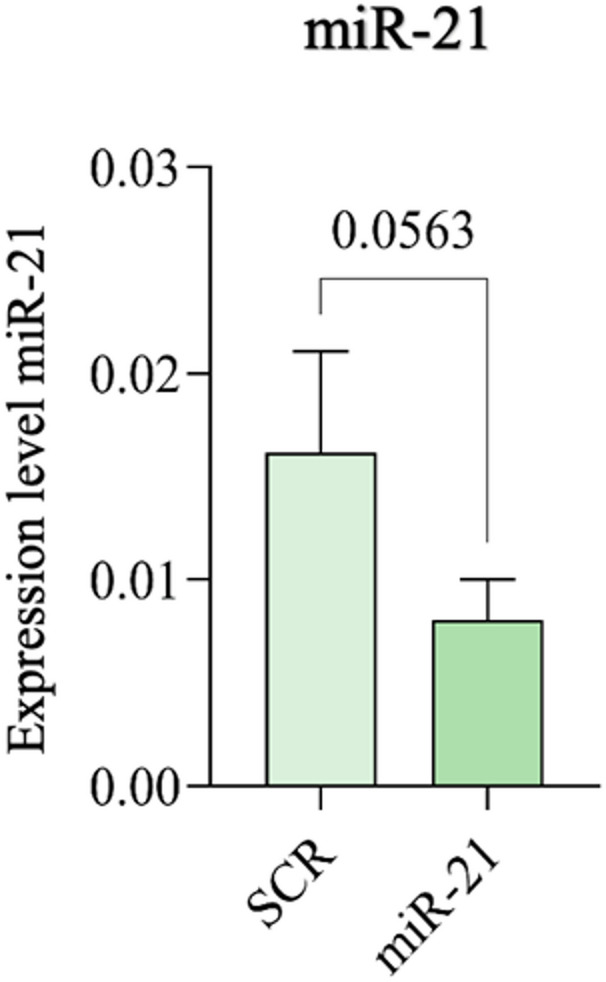
miR-21 expression in T24 cells following CRISPR/Cas9-mediated editing. miR-21 expression levels were quantified by RT-qPCR in T24 cells treated with CRISPR/Cas9 RNP complexes containing sgRNA targeting miR-21 and compared to the scramble control group. Relative expression was calculated using the 2^−ΔΔCt^ method and normalized to RNU48. Data are presented as mean ± standard deviation from at least three independent biological experiments performed in technical duplicates. Statistical analysis was performed using Student’s t-test

miR-21 expression was evaluated by RT-qPCR in cells treated with the CRISPR/Cas9 system containing an sgRNA targeting miR-21, compared to the scramble control group. A reduction in miR-21 expression levels was observed in treated cells; however, this difference did not reach statistical significance (*p* = 0.0563), indicating a limited or heterogeneous effect of the editing approach under the experimental conditions (Fig. 1).

### Regulation of target genes and PD-L1 expression

**Fig. 2:**
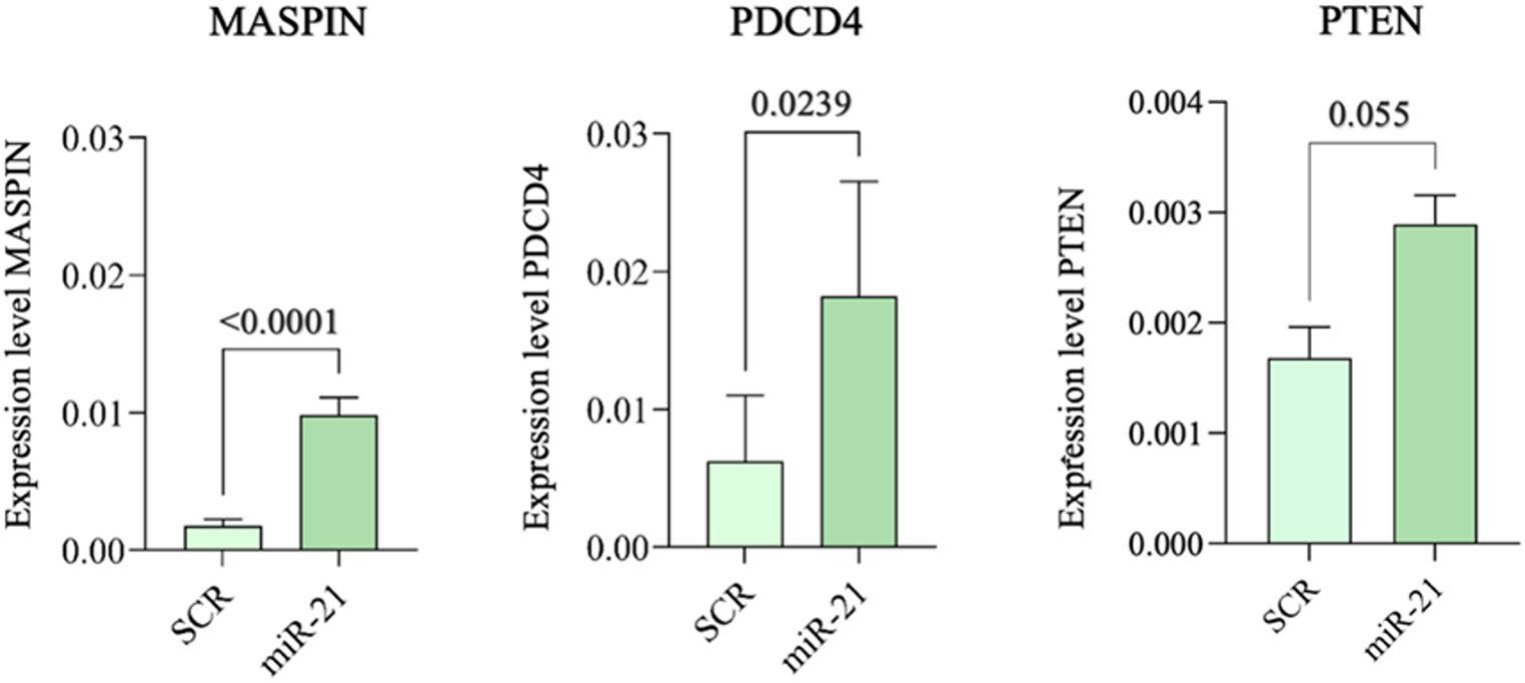
Expression of miR-21 target genes in T24 cells following CRISPR/Cas9-mediated modulation. Expression levels of MASPIN, PDCD4, and PTEN were evaluated by RT-qPCR in T24 cells treated with CRISPR/Cas9 targeting miR-21 and compared to the scramble control group. Gene expression was normalized to B2M and analyzed using the 2^−ΔΔCt^ method. Data are presented as mean ± standard deviation from at least three independent biological experiments performed in technical duplicates. Statistical analysis was performed using Student’s t-test. Exact p-values are indicated in the graphs

**Fig. 3:**
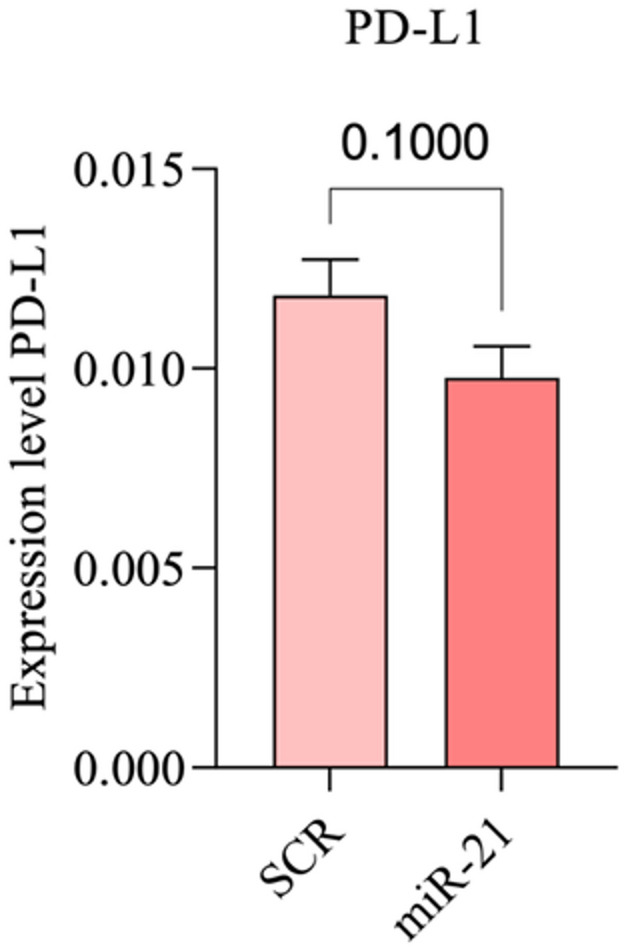
PD-L1 gene expression following miR-21 modulation. RT-qPCR quantification of PD-L1 expression in T24 cells treated with CRISPR/Cas9 targeting miR-21 and in the scramble control group. Expression levels were normalized to B2M and calculated using the 2^−ΔΔCt^ method. Data are presented as mean ± standard deviation from at least three independent biological experiments performed in technical duplicates. Statistical analysis was performed using Student’s t-test

Despite the lack of statistically significant miR-21 downregulation, significant modulation of its molecular targets was observed. Treated cells exhibited increased expression of the tumor suppressor genes MASPIN (*p* < 0.0001) and PDCD4 (*p* = 0.0239), as well as a trend toward increased PTEN expression (*p* = 0.055), compared to the control group (Fig. 2). These findings suggest functional downstream effects consistent with partial inhibition of miR-21 activity. No significant reduction in PD-L1 expression was observed (*p* = 0.1000) (Fig. 3).

### Effect of miR-21 modulation on cell migration

**Fig. 4:**
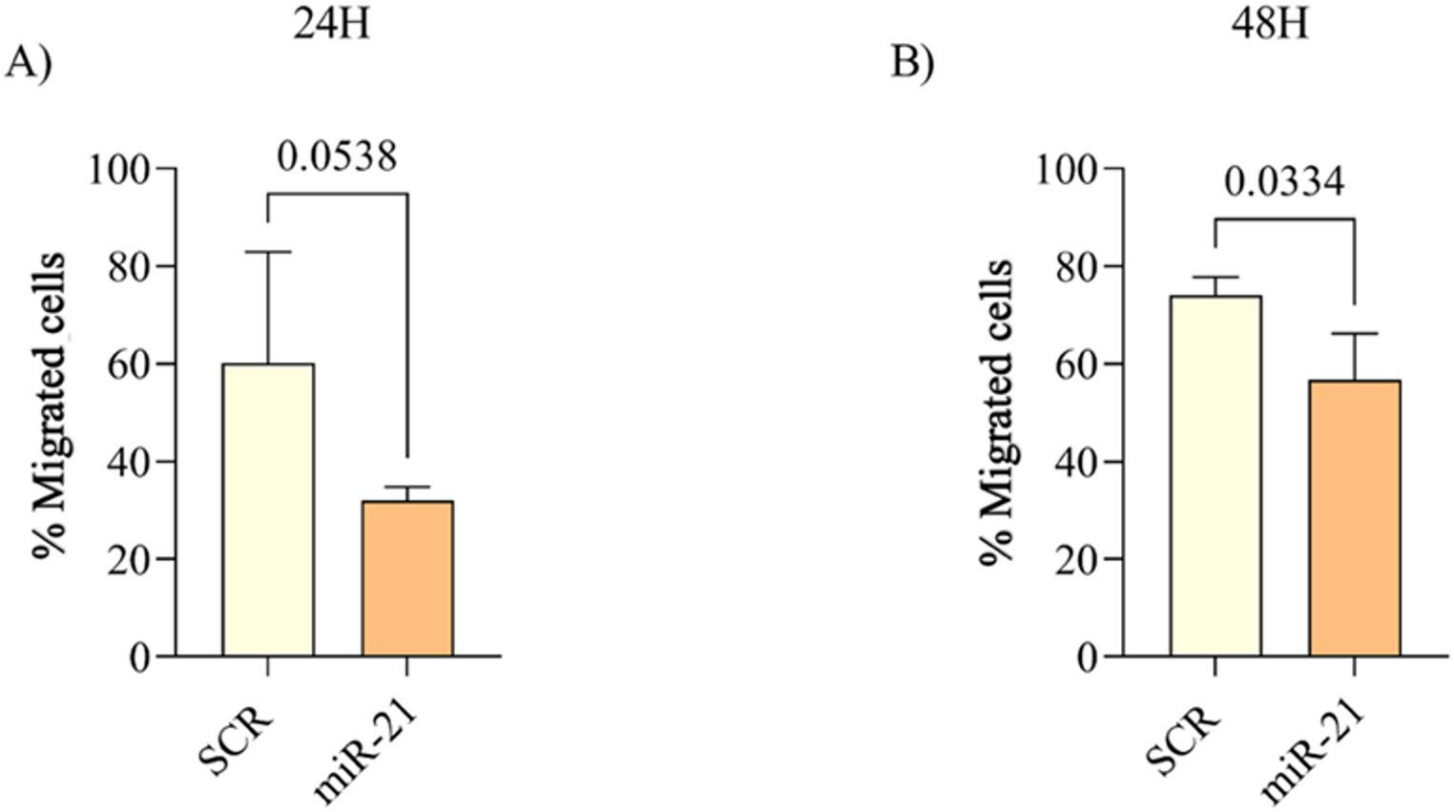
Quantitative analysis of cell migration. Quantitative analysis of wound closure in T24 cells treated with CRISPR/Cas9 targeting miR-21 or scramble control at 0, 24, and 48 h. Migration was calculated as [(D_initial − D_final) / D_initial] × 100. Data are presented as mean ± standard deviation from at least three independent biological experiments performed in technical duplicates. Statistical comparisons between groups were performed using Student’s t-test

**Fig. 5:**
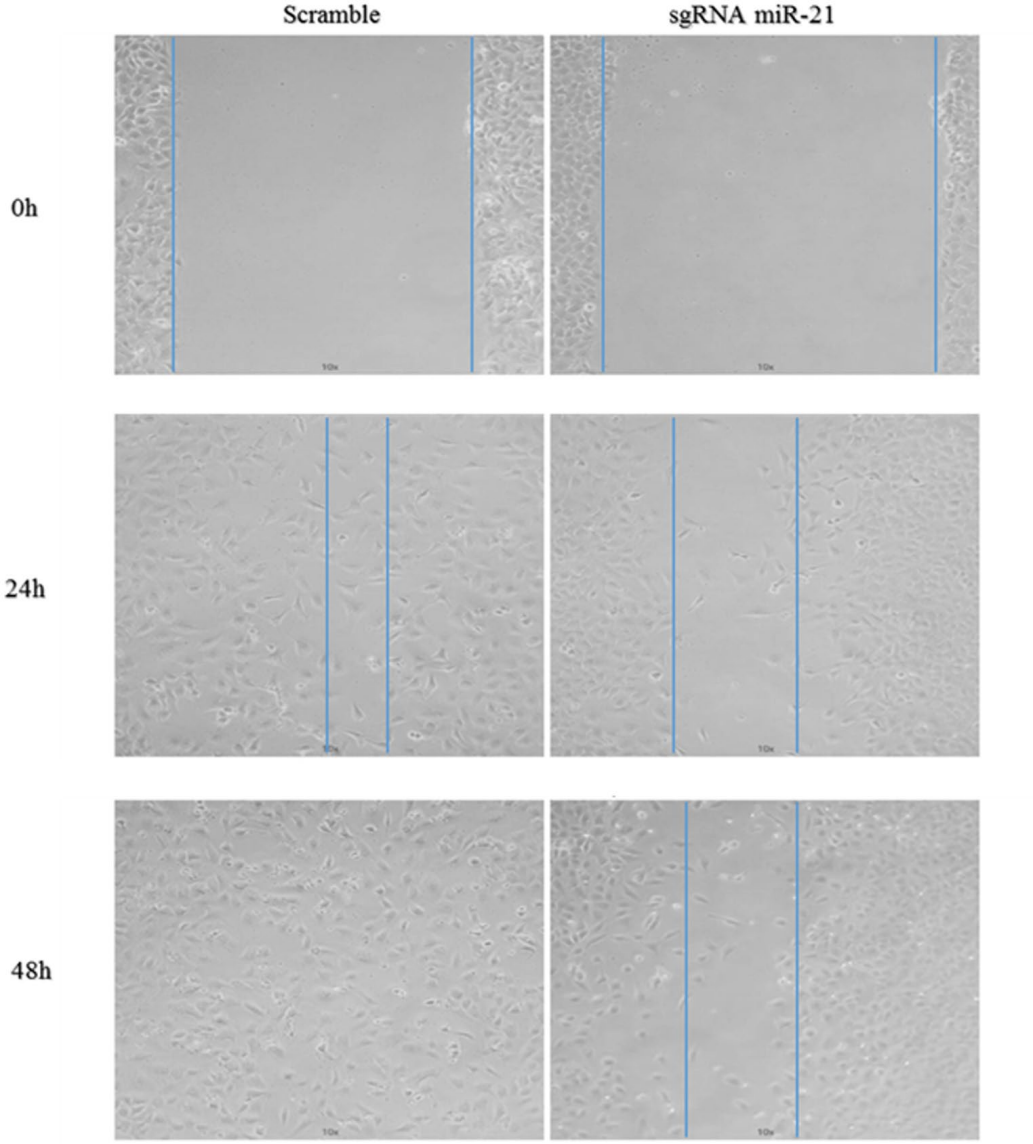
Representative images of the cell migration assay. Representative images of wound closure in T24 cells transfected with scramble control or sgRNA targeting miR-21 at 0, 24, and 48 h after scratch induction. Dashed lines indicate wound boundaries. Images are representative of independent biological experiments

Cell migratory capacity was assessed using a migration assay. After 24 h, the results indicated a trend toward reduced cell migration in the treated group (*p* = 0.0538). After 48 h, this reduction became statistically significant (*p* = 0.0334) (Fig. 4). Qualitative image analysis confirmed the quantitative results (Fig. 5).

### Evaluation of invasive potential

**Fig. 6:**
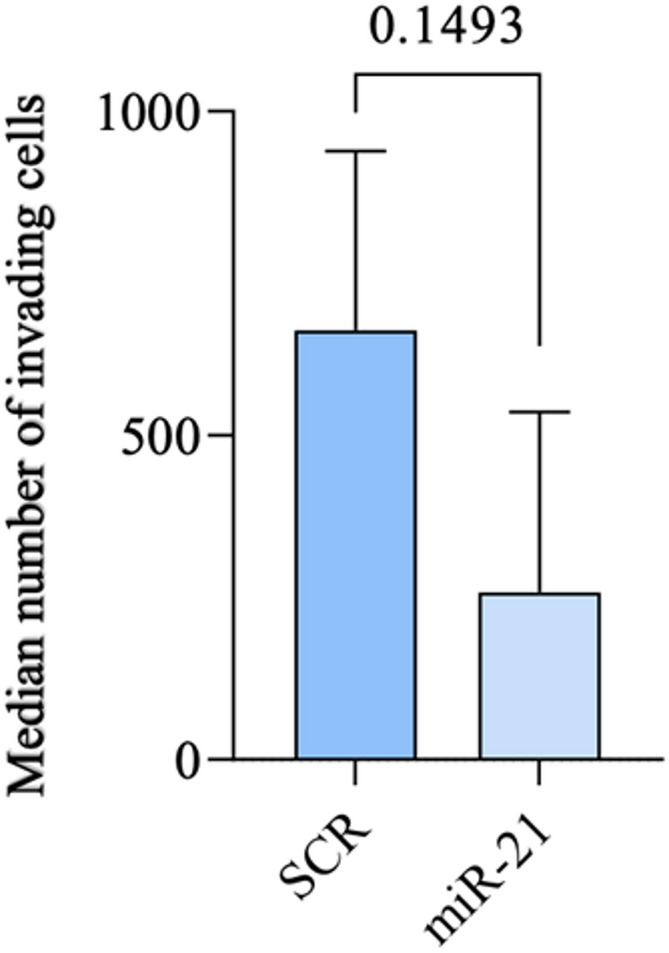
Quantitative analysis of cell invasion. Quantification of invading T24 cells following treatment with CRISPR/Cas9 targeting miR-21 or scramble control. Invasive cells were quantified 48 h after seeding in Matrigel™ invasion chambers. Data are presented as mean ± standard deviation from at least three independent biological experiments performed in technical duplicates. Statistical analysis was performed using Student’s t-test

**Fig. 7:**
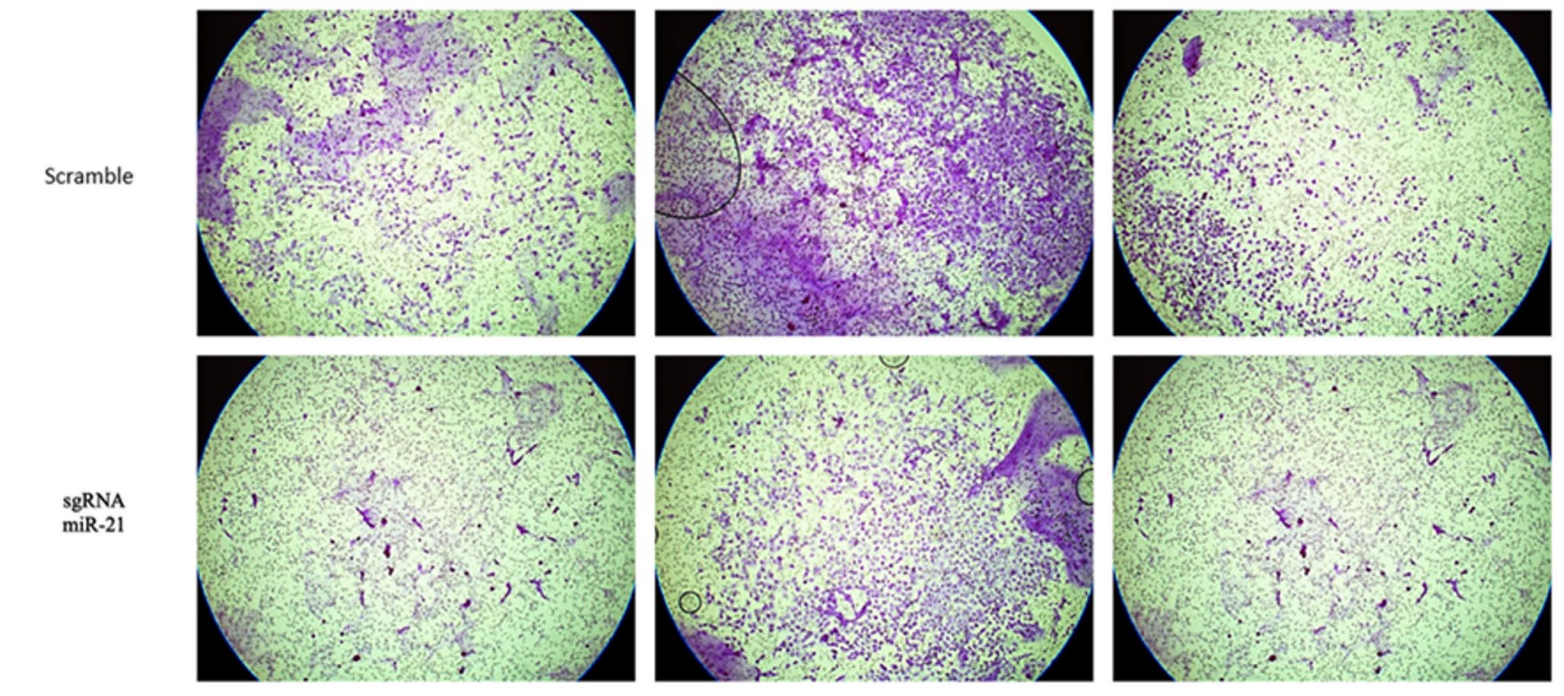
Representative images of the Matrigel™ invasion assay. Representative images of invading T24 cells following treatment with CRISPR/Cas9 targeting miR-21 or scramble control. Cells that migrated through the Matrigel-coated membrane were fixed, stained with crystal violet, and visualized under light microscopy. Images are representative of independent biological experiments

Invasive potential was assessed using Matrigel™ invasion chambers. Quantitative analysis showed no statistically significant difference between treated and control groups (*p* = 0.1493) (Fig. 6). Although qualitative observations suggested a lower density of invading cells in the treated group, these findings were not supported by quantitative analysis and should therefore be interpreted with caution (Fig. 7).

### Validation of transfection by immunofluorescence

**Fig. 8:**
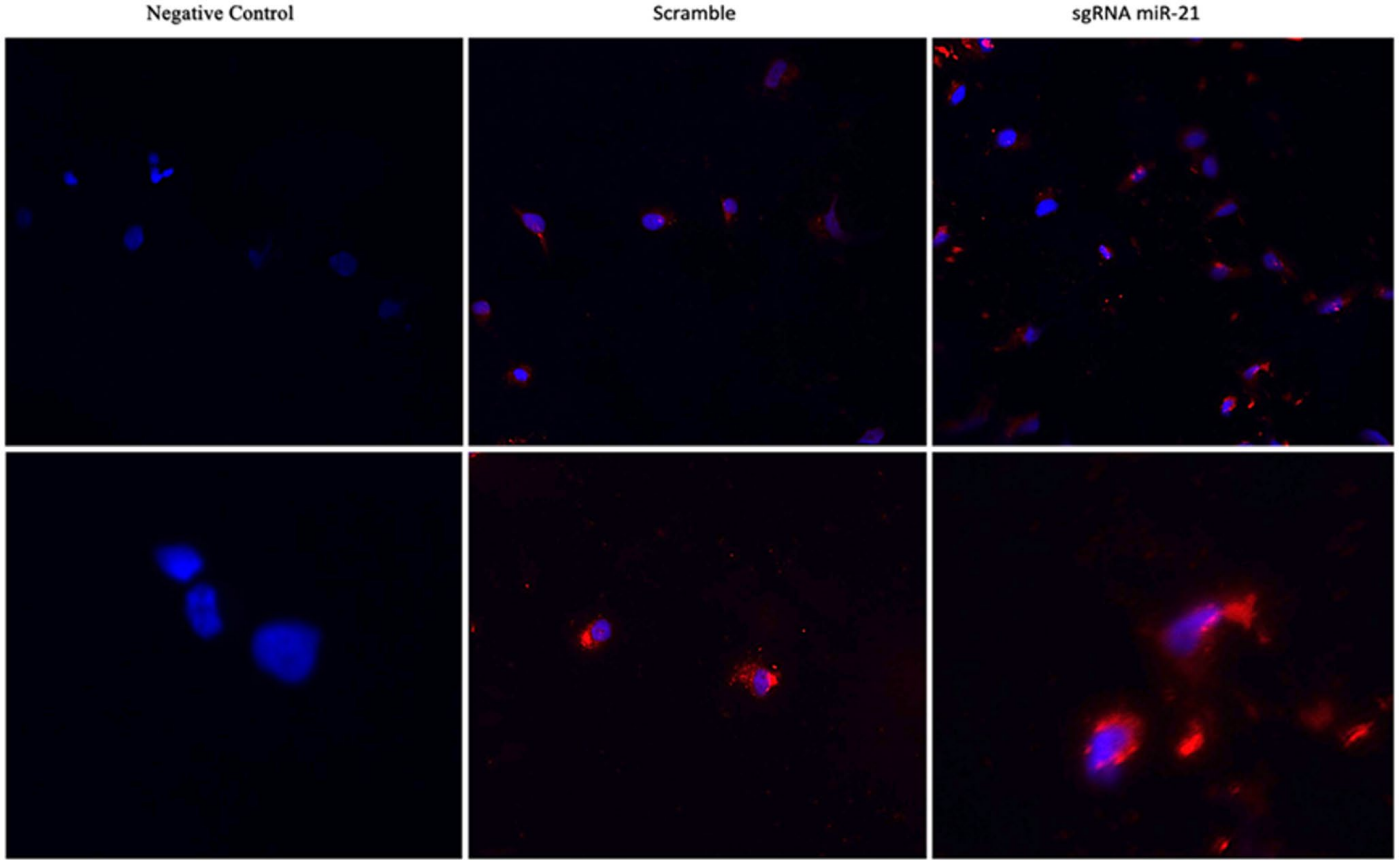
Immunofluorescence analysis of Cas9 delivery. Immunofluorescence detection of Cas9 protein in T24 cells transfected with CRISPR/Cas9 RNP complexes containing scramble control or sgRNA targeting miR-21. Cas9 protein is shown in red and nuclei are counterstained with DAPI (blue). The negative control, performed without the primary antibody, confirmed staining specificity. Images are representative of independent experiments

Immunofluorescence analysis was performed to evaluate Cas9 protein expression in T24 cells following treatment with the CRISPR/Cas9 system. Fluorescent signal compatible with Cas9 protein expression was detected in transfected cells from both the scramble control group and the group treated with sgRNA targeting miR-21 (Fig. 8).

The specificity of staining was confirmed by the absence of fluorescent signal in the negative control, in which the primary antibody was omitted, supporting the specific detection of Cas9 protein in transfected cells.

## Discussion

Modulation of miR-21 by CRISPR/Cas9 in T24 cells was associated with molecular and functional alterations. The use of the CRISPR/Cas9 system through RNP complex delivery represents a transient and highly controllable genome-editing strategy, characterized by rapid activity and a lower risk of off-target effects compared to vector-based approaches [28, 31, 32]. Detection of Cas9 protein by immunofluorescence confirmed system internalization, supporting effective delivery of the editing machinery. However, Cas9 detection and RT-qPCR analysis represent indirect evidence of modulation and do not constitute direct confirmation of genomic editing. Although modulation of miR-21 expression did not reach statistical significance, a trend toward reduction was observed, suggesting partial and possibly heterogeneous editing efficiency. This result may be explained by cellular heterogeneity and the absence of clonal selection, indicating that only a fraction of the cells was effectively edited, thereby reducing the overall impact of the observed modulation [28].

Even in a scenario of partial modulation, a significant increase in the expression of tumor suppressor genes MASPIN and PDCD4, as well as a trend toward increased PTEN expression, supporting functional derepression of miR-21 downstream targets despite incomplete knockdown. These findings are consistent with the role of miR-21 as an oncomiR, acting in the post-transcriptional repression of multiple genes involved in the regulation of tumor growth and progression [15, 16]. In this context, restoration of PDCD4 is particularly relevant, as miR-21 directly regulates its expression. Functionally, PDCD4 acts as a negative regulator of translation and of pro-invasive pathways, such as AP-1 transcription factor activity, which may partly explain the effects observed in functional assays [19–21]. In addition, consistency with previous reports, such as those described by Camargo et al. (2023), suggests that this mechanism may be conserved across different tumor contexts [27].

Similarly, an inverse relationship between miR-21 and MASPIN has been described in BC. Elevated miR-21 levels are associated with reduced MASPIN expression and a more aggressive tumor phenotype, including advanced stage, higher histological grade, lymph node metastasis, and poorer prognosis [18]. Loss of MASPIN is directly linked to increased invasiveness and tumorigenesis. Mechanistically, this gene may act as an inhibitor of HDAC1, regulating targets involved in cell proliferation and invasion, such as p21, cyclin D1, and MMP9 [18, 22]. Thus, the increased expression of MASPIN observed after miR-21 modulation in our model is consistent with derepression of this regulatory axis, potentially contributing to the modulation of pathways controlling invasion and tumor growth.

Another relevant finding was the trend toward increased PTEN, a direct target of miR-21 and a negative regulator of the PI3K/AKT pathway, which is involved in cell proliferation, survival, and tumor metabolism [23, 24]. This finding suggests partial restoration of this signaling axis following miR-21 modulation. Based on evidence from other tumor models, in which PTEN loss is associated with activation of the PI3K/AKT/mTOR pathway and increased PD-L1 expression as a mechanism of immune evasion [25, 26], it was hypothesized that increased PTEN could reduce PD-L1 levels. However, no significant changes in PD-L1 expression were observed, indicating that, in this model of high-grade bladder cancer, its regulation is likely multifactorial and not predominantly driven by the miR-21/PTEN axis alone [26].

In the migration assay, miR-21 modulation led to reduced cell motility, with a significant difference observed after 48 h, supporting a time-dependent functional impact consistent with its pro-migratory role described in other tumor types [18, 22]. In bladder cancer, this effect may be related to derepression of targets such as MASPIN, whose function involves regulation of cell–extracellular matrix interaction and cytoskeletal dynamics, processes directly linked to migration [18, 22, 27]. However, wound-healing assays may also be influenced by proliferation-related effects, and therefore the migration findings should be interpreted cautiously. Furthermore, migration was evaluated using a wound-healing assay rather than a Boyden/transwell migration system. Although widely used, wound-healing assays may be influenced by proliferation-related effects. Therefore, additional migration analyses using non-Matrigel Boyden chamber systems could provide complementary information regarding cell motility independent of cell proliferation.

In the invasion assay, a non-significant trend toward reduced invasive capacity was observed compared to the control group. miR-21 negatively regulates PDCD4, and its reexpression may impact invasion by inhibiting eIF4A activity and, consequently, the translation of proteins involved in tumor progression [19–21]. In addition, MASPIN also contributes to this process by modulating the extracellular matrix and protease activity [18, 27]. The observed trend may reflect this combined action, but the lack of statistical significance suggests a modest effect size or insufficient editing efficiency to produce a robust phenotypic change. Factors such as assay variability and the presence of non-edited cells following CRISPR/Cas9 may also have attenuated the observed effect.

Although the results are consistent with the proposed hypothesis, some limitations should be considered. Editing efficiency was assessed exclusively by RT-qPCR, without validation by sequencing, which limits direct confirmation of genomic alterations. In addition, no sequencing-based methods, such as amplicon sequencing or mismatch cleavage assays, were performed to directly confirm editing at the miR-21 locus. Furthermore, the absence of clonal selection and the use of a single cell line (T24) limit the generalizability and mechanistic interpretation of the findings. Additional validation using other urothelial carcinoma models would strengthen the biological applicability of the results. Moreover, protein-level validation of downstream targets and complementary functional assays evaluating proliferation, viability, apoptosis, or clonogenic potential were not performed and could further clarify the biological impact of miR-21 modulation. Despite these limitations, the results demonstrate that even partial modulation of miR-21 was associated with increased expression of PTEN, PDCD4, and MASPIN, genes involved in tumor suppression. The reduction in migration and the trend toward decreased invasion reinforce the idea that miR-21 modulation interferes with processes relevant to tumor behavior.

## Conclusion

CRISPR/Cas9-mediated modulation targeting miR-21, delivered via RNP, despite not significantly reducing miR-21 expression, was associated with measurable molecular and functional alterations in T24 bladder cancer cells. The increased expression of PDCD4 and MASPIN, as well as a trend toward increased PTEN expression, suggests partial reactivation of tumor suppressor pathways even with incomplete modulation. The reduction in cell migration, in contrast to the non-significant effect on invasion, suggests that these processes may respond differently to miR-21 modulation. Furthermore, the absence of changes in PD-L1 despite the trend toward increased PTEN expression points to a complex and context-dependent regulatory network. Overall, these findings reinforce the potential involvement of miR-21 in tumor progression and support further investigation of miR-21-targeted approaches in bladder cancer. However, optimization of modulation efficiency and validation using complementary molecular approaches, protein-level analyses, and additional experimental models are required.

## Supplementary Information

Below is the link to the electronic supplementary material.

Supplementary Material 1

## Data Availability

The datasets generated and/or analyzed during the current study are available from the corresponding author on reasonable request.
