# Supplementary material for: CRISPR/Cas9-mediated miR-21 editing in high-grade urothelial carcinoma cells and its biological effects
Source: Mol Biol Rep. 2026 Jun 19;53(1):973. doi: 10.1007/s11033-026-12129-7 (PMC13282212; doi:10.1007/s11033-026-12129-7)
Supplement: Supplementary file 1 — Supplementary Material 1 [file 11033_2026_12129_MOESM1_ESM.docx]

| **Target** | **Assay ID** | **Manufacturer** |
| --- | --- | --- |
| MASPIN | Hs00985283_m1 | Applied Biosystems |
| PDCD4 | Hs00377253_m1 | Applied Biosystems |
| PTEN | Hs02621230_s1 | Applied Biosystems |
| PD-L1 | Hs00204257 | Applied Biosystems |
| miR-21 | 000397 | Applied Biosystems |
| RNU48 | HS00984230 | Applied Biosystems |
| B2M | Hs00187842_m1 | Applied Biosystems |

**Supplementary Table 1.** TaqMan™ assay identification codes used for RT-qPCR analysis of target genes, endogenous controls, and miR-21 expression in T24 urothelial carcinoma cells. All assays were obtained from Applied Biosystems.
